# Short-term pacing in the mouse alters cardiac expression of connexin43

**DOI:** 10.1186/1472-6793-8-8

**Published:** 2008-05-06

**Authors:** Andrianos Kontogeorgis, Riyaz A Kaba, Eunice Kang, Jonathan E Feig, Pritha P Gupta, Marc Ponzio, Fangyu Liu, Michael J Rindler, Andrew L Wit, Edward A Fisher, Nicholas S Peters, David E Gutstein

**Affiliations:** 1 Leon H. Charney Division of Cardiology, Department of Medicine, New York University School of Medicine, New York, NY, USA; 2 Department of Cell Biology, New York University School of Medicine, New York, NY, USA; 3 Department of Pharmacology, Columbia University College of Physicians and Surgeons, New York, NY, USA; 4 Department of Cardiology, St Mary's Hospital, Imperial College London, UK

## Abstract

**Background:**

Cardiac insults such as ischemia, infarction, hypertrophy and dilatation are often accompanied by altered abundance and/or localization of the connexin43 gap junction protein, which may predispose towards arrhythmic complications. Models of chronic dyssynchronous cardiac activation have also been shown to result in redistribution of connexin43 in cardiomyocytes. We hypothesized that alterations in connexin43 expression and localization in the mouse heart might be induced by ventricular pacing over a short period of time.

**Results:**

The subdiaphragmatic approach was used to pace a series of wild type mice for six hours before the hearts were removed for analysis. Mice were paced at 10–15% above their average anesthetized sinus rate and monitored to ensure 1:1 capture. Short-term pacing resulted in a significant reduction in connexin43 mRNA abundance, a partial redistribution of connexin43 from the sarcolemma to a non-sarcolemmal fraction, and accumulation of ubiquitinated connexin43 without a significant change in overall connexin43 protein levels. These early pacing-induced changes in connexin43 expression were not accompanied by decreased cardiac function, prolonged refractoriness or increased inducibility into sustained arrhythmias.

**Conclusion:**

Our data suggest that short-term pacing is associated with incipient changes in the expression of the connexin43 gap junction, possibly including decreased production and a slowed rate of degradation. This murine model may facilitate the study of early molecular changes induced by pacing and may ultimately assist in the development of strategies to prevent gap junction remodeling and the associated arrhythmic complications of cardiac disease.

## Background

Sudden cardiac death is a common and tragic complication of heart disease that affects more than 400,000 Americans annually [1]. The pathophysiology of myocardial remodeling, at functional and molecular levels, results in a substrate ripe for arrhythmic complications [2]. Remodeling of connexin43 (Cx43) gap junction distribution and expression has been described in ischemia, infarction, hibernating myocardium and dilated cardiomyopathy [3-6] and is a potentially significant contributor to the arrhythmogenicity of cardiac disease [7-9]. Indeed, we have observed that decreased expression and mislocalization of Cx43 to the lateral borders of myocytes in the peri-infarct zone (a process termed "structural gap junction remodeling") was found to correspond spatially to the central common pathway of figure-of-eight reentrant ventricular tachycardia circuits [6].

Chronically altered ventricular activation, such as that induced in canine models using pacing or radiofrequency ablation of the left bundle branch, has been associated with focal structural gap junction remodeling [10,11]. Areas of gap junction remodeling observed in the dog heart after 21 days of pacing were localized exclusively to regions in close proximity to the pacing electrode [11]. Since less than an hour of ischemia-reperfusion in an isolated rat heart is required to demonstrate intracellular redistribution of Cx43 [12], we predicted that pacing-induced gap junction remodeling might occur over a short term.

To test our hypothesis, we adapted the subdiaphragmatic approach for programmed electrical stimulation in the mouse heart [13] to allow for six hours of single lead ventricular pacing at rates just above those of sinus rhythm. We used a murine model since it might facilitate molecular studies of remodeling by eventually allowing for the use of transgenic models to elucidate mechanisms. Pacing via the subdiaphragmatic approach was associated with significant mechanical dyssynchrony. After six hours of pacing, we observed a significant decrease in Cx43 mRNA levels, evidence of intracellular redistribution of Cx43 protein, and an accumulation of ubiquitinated Cx43 in the paced hearts. Our findings were consistent with decreased production and disrupted degradation of Cx43, possibly accounting for preserved total Cx43 protein levels despite decreased mRNA expression and reduced membrane immuno-localization of Cx43 after pacing.

These data suggest that alterations in the expression and distribution of Cx43 occur after limited exposure to pacing. Changes in Cx43 expression patterns may contribute to increased arrhythmogenicity in the presence of additional insults to the heart. Since gap junction alterations may be preventable or reversible, elucidating the pathways involved may ultimately allow for the pharmacologic targeting of intermediaries in those pathways and the prevention of arrhythmic complications.

## Methods

### Pacing Procedure

Mice aged 3 – 4 months were used for these experiments. All studies were performed in accordance with the regulations of the Institutional Animal Care and Use Committee of the New York University School of Medicine (New York, NY). The C57BL/6J strain was used for all experiments except for immunoblotting of ventricular lysates and quantification of mRNA expression, for which CD-1 mice were used. Mice were anesthetized with inhaled isoflurane (4 vol% induction, 1.5 vol% maintenance; Baxter, Deerfield, IL) and immobilized on a heating pad set to 37°C. Electrocardiographic signals from limb leads were monitored during the experiment and recordings were obtained prior to the initiation of pacing, during pacing (to document 1:1 capture) and following cessation of pacing.

Following recording of the baseline electrocardiogram, a 1 cm midline incision was made in the epigastric region. A custom-designed UE-GM1 cardiac stimulating electrode with a 200 μm monopolar platinum tip (Frederick Haer & Co., Bowdoinham, ME) mounted on a micromanipulator (Fine Science Tools, Inc., North Vancouver, BC, Canada) was inserted through the diaphragm directly into contact with the surface of the beating right ventricle. Electrode contact was ensured by monitoring the electrocardiographic activity. Pacing was performed with a Model 2352 Programmable Stimulator (Medtronic, Minneapolis, MN). Output was set at twice the stimulating threshold in all animals, with a pulse width of 1.0 ms. Pacing cycle lengths were set and maintained at 10–15% above the underlying heart rate, to ensure 100% capture for 6 hours. To serve as controls, age-, sex- and strain-matched mice were prepared and anesthetized with the pacing electrode placed into contact with the heart identically to the paced cohort, but the programmable stimulator was not switched on ("sham-paced").

### Electrocardiographic Recordings

Electrocardiographic signals were recorded as previously described. Electrocardiographic intervals were calculated from leads I, II and III recorded at the beginning of each study and after the completion of the pacing protocol.

### Echocardiography

Echocardiography was performed according to a previously described protocol [14] on paced and sham-paced mice (n = 6 in each group), prior to and during or immediately following six hour pacing and sham protocols. Mice were imaged using a Philips HDI 5000 echocardiography machine equipped with a 15 MHz linear probe.

### In Vivo Electrophysiology

Programmed electrical stimulation (PES) was carried out in paced and sham-paced mice at baseline and after the pacing protocol as described previously [13]. The PES protocol consisted of trains of eight beats at pacing cycle lengths of 100 ms and 80 ms, followed by single extrastimuli for the determination of ventricular effective refractory period (VERP) and double extrastimuli to assess for inducible arrhythmias. PES data represent the average of two trials at separate sites on each heart.

### Tissue Handling and Immunolabeling

Following the pacing procedure, hearts destined for immunostaining were rapidly excised and frozen in Tissue Tek OCT compound (Sakura Finetek USA, Inc., Torrance, CA). Five micron-thick sections were cut in an HM 560 cryostat (Microm, Walldorf, Germany) at -20°C, placed onto Superfrost/Plus microscope slides (Fisher Scientific, Pittsburgh, PA) and fixed in acetone. We selected frozen sections to evaluate the left ventricle from basal, mid-ventricular, and apical regions. We defined the basal region as those sections proximal to the papillary muscles; mid-ventricular regions were those sections of LV with clearly identified papillary muscles; and apical regions were distal to the papillary muscles. We also evaluated apical and basal sections of the right ventricle.

Sections were blocked and then double-stained with a custom-made rabbit polyclonal anti-Cx43 antibody [15] at 1:1000 and wheat germ agglutinin to visualize myocyte borders as previously described [16]. For visualization of cadherin staining, frozen sections were incubated with a rabbit anti-pan-cadherin antibody (1:5000; Sigma), followed by a Texas Red-conjugated goat anti-rabbit secondary antibody (Jackson ImmunoResearch Laboratories) and a FITC-conjugated anti-Cx43 antibody. Stained sections were visualized on an Axioskop 2 Plus fluorescence microscope. Images were collected using uniform exposure settings for each staining run on an Axiocam camera with AxioVision 4.30 software (Carl Zeiss, Munchen-Hallbergmoos, Germany).

### Quantification of Cx43 and Cadherin Immunosignal

Images of immuno-stained sections from paced and sham-paced hearts were obtained from blinded slides and digitally archived for offline analysis. Blinded image files were uniformly thresholded by eliminating signal-free areas above and below the distribution of intensity values using the histogram function on Adobe Photoshop. Digital image processing was then performed according to previously established techniques [17], again in a blinded fashion, with NIH Image J to determine Cx43 and cadherin signal area as a percentage of total tissue area, as well as Cx43 plaque size and the number of Cx43 plaques per 40× field.

### Immunoblotting and Densitometry

For the evaluation of total protein levels by immunoblotting, endocardial ventricular tissue from paced and sham-paced hearts was prepared by Dounce homogenization in lysis buffer supplemented with Complete protease inhibitor cocktail (Roche, Mannheim, Germany). Endocardial samples were prepared by mounting the excised LV free wall in an OCT block, separating the endocardial, mid-myocardial and epicardial regions by sectioning on an HM 560 cryostat and homogenizing as above. The inner third of the LV free wall was considered the endocardial region, middle third mid-myocardial and outer third epicardial. Protein concentrations were determined by Bradford assay performed in triplicate and equal loading was confirmed with coomassie staining. Proteins were electrophoresed on 10% SDS-PAGE gels and transferred onto nitrocellulose blots (Bio-Rad Laboratories, Hercules, CA). Immunoblots were blocked followed by incubation with appropriate primary antibodies directed against Cx43, cadherin (see above), Cx45 [18], Cx40 (Alpha Diagnostics, San Antonio, TX) and GAPDH (Chemicon/Millipore, Billerica, MA). HRP-conjugated secondary antibody (Santa Cruz Biotechnology, Santa Cruz, CA) was then applied, followed by HyGlo chemiluminescent processing (Denville Scientific, Metuchen, NJ) and autoradiography. At least two separate experiments were quantified by scanning the autoradiograms on a Bio-Rad Gel Doc GS 800 and calculating band intensity using Quantity One software (Bio Rad, Hercules, CA). Connexin band intensities were normalized to the relative intensity of the corresponding GAPDH band for each sample. Results were expressed as a percentage of matched sham-paced controls.

### Fraction Preparation and Immunoprecipitation

To determine sarcolemmal and non-sarcolemmal Cx43 concentrations, fractionation of samples was performed as described [15,19]. Briefly, heart samples were Dounce homogenized, centrifuged at 500 × g for 10 minutes to remove insolubles and layered over a 45% sucrose cushion. After centrifuging at 7000 × g for 20 minutes, the supernatant (non-sarcolemmal fraction) was separated from the cloudy layer immediately overlying the sucrose (sarcolemmal fraction). Protein concentrations in each fraction were determined by Bradford assay performed in triplicate and equal loading was confirmed with coomassie staining. The resulting fractions were analyzed by SDS-PAGE and Western blotting.

For immunoprecipitation, 50 μg of total heart lysate was incubated with polyclonal anti-Cx43 antibody. After addition of protein A-agarose-immobilized protein beads (Roche) to the samples, the protein A suspension was centrifuged at 5000 × g and the supernatant was removed. The protein A beads were washed in IP buffer and resuspended in loading buffer prior to incubation at 100°C and analysis by SDS-PAGE. The resulting blots were incubated with a monoclonal antibody directed against ubiquitin (FK2, Biomol) and processed as above.

### RNA Isolation and Quantitative Real-Time PCR (qRT-PCR)

To isolate total RNA, the LV free walls of paced and sham-paced hearts were excised, mounted in OCT and snap frozen in liquid nitrogen. Endocardial and epicardial thirds were collected by sectioning through the LV free wall as described above. RNA was isolated using Trizol (Invitrogen, Carlsbad, CA) according to the manufacturer's recommendations. RNA quality was verified with the Agilent 2100 Bioanalyzer (Agilent Technologies, Santa Clara, CA). The concentration of RNA was determined by the Quant-iT RiboGreen RNA Assay Kit (Invitrogen, Carlsbad, CA). Real time quantitative PCR was performed using the ABI Prism 7700 Sequence Detection System (Applied Biosystems, Foster City, CA). Cx43 primer sequences, located in the 3' untranslated region, were as follows: forward primer sequence was 5'-GTGCCGGCTTCACTTTCATTAAG-3'; reverse primer sequence was 5'-ACTGACCTCGCGGAACC-3'; probe sequence was 5'-TTTCTCTCCACGGGTCT-3'. All data were normalized to Cyclophilin A (primer sequences as per [20]) and expressed as fold change compared to the sham-paced controls. Negative controls were performed for each of the samples, in which reverse transcriptase was not added prior to RNA quantification. Results per animal represent the mean of six measurements of each endocardial and epicardial sample.

### Statistics

Data are expressed as mean ± SEM. Quantitative immunofluorescence data were compared between groups with ANOVA using StatView (SAS Institute, Inc., Cary, NC). Data from electrocardiography, echocardiography, PES, immunoblot densitometry and qRT-PCR were compared between groups with unpaired two-tailed t-tests (Microsoft Excel). Electrocardiographic indices, echocardiographic measurements and ERP values obtained during or after the pacing protocol were compared to baseline measurements with paired two-tailed t-tests (Microsoft Excel). P < 0.05 was considered statistically significant.

## Results

### Stimulating Electrode is Consistently Positioned on the Right Ventricle

**Figure 1:**
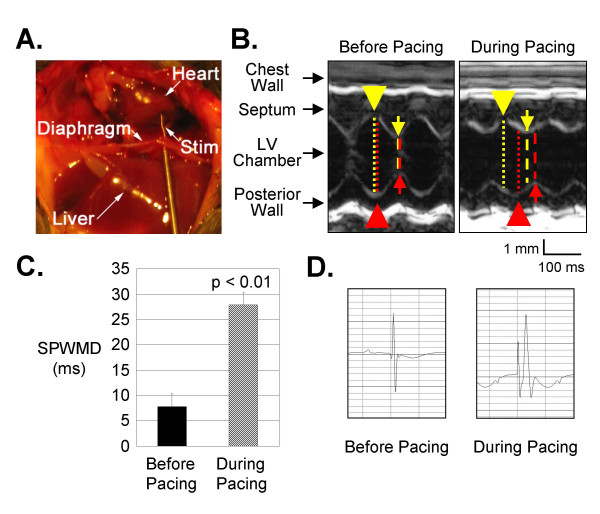
**Pacing from the Subdiaphragmatic Approach is Associated with Mechanical Dyssynchrony**. **A) **For illustrative purposes only, a mid-line sternotomy was performed after the pacing experiment to demonstrate that the tip of the stimulating electrode is positioned at the epicardial surface of the right ventricle. **B) **M-mode echocardiography reveals dyssynchrony of ventricular contraction during subdiaphragmatic pacing with a substantially greater delay in the time between septal (yellow arrow) and posterior wall thickening (red arrow) compared to non-paced baseline. **C) **Septal-to-Posterior Wall Motion Delay (SPWMD), a measurement of the time interval between septal and posterior wall systolic thickening as demonstrated in Panel A, is significantly prolonged during pacing in comparison to the SPWMD measured prior to initiation of pacing. **D) **A surface electrocardiogram recorded during pacing demonstrates a wide and aberrantly conducted paced complex in comparison to a sinus beat recorded before pacing.

For ease of pacing and reliability, we used the subdiaphragmatic approach, which allows for placement of the stimulating electrode on the heart without necessitating sternotomy, thoracotomy, mechanical ventilation or manipulation of the vasculature [13]. For the investigation of probe placement after the pacing protocol was completed, a midline sternotomy was performed at the end of four consecutive experiments. In all four animals examined in this fashion, the stimulating electrode traversed the diaphragm and made contact with the right ventricular (RV) surface without puncturing the heart (Figure 1A). Local structures (including the abdominal viscera, diaphragm and lungs) were not disrupted by the electrode. Thus, the pacing model employed in this study can be considered as single-lead RV epicardial pacing.

### Threshold is Unchanged after Pacing Protocol

Since our objective was to model altered activation rather than induce tissue damage [21], we limited the stimulator output to approximately twice the stimulating threshold (mean stimulator output was 0.366 ± 0.011 mA). The average threshold prior to pacing was 0.165 ± 0.007 mA (n = 65). In a subset of eight mice, the threshold was determined after pacing. In this group the average post-pacing threshold was 0.162 ± 0.026 mA (p = NS compared to pre-pacing values). The unchanged threshold values after pacing underscore the stability of the experimental preparation.

### Short-Term Pacing Is Associated with Mechanical Dyssynchrony

To determine whether short-term pacing influenced synchrony of cardiac contraction, we examined mice prior to and during pacing using echocardiography. The septal-to-posterior wall motion delay (SPWMD), an echocardiographic index of dyssynchronous contraction [22,23], increased significantly from 7.8 ± 2.5 ms before pacing to 27.9 ± 2.4 ms during pacing (p < 0.01; n = 6 each; Figure 1B and 1C). Delay in onset of systolic wall thickening measured from the septal to the posterior walls also increased significantly during pacing (12.8 ± 1.1 ms at baseline vs. 31.6 ± 1.4 ms during pacing, p < 0.001). As expected, surface electrocardiograms demonstrated substantial widening of the QRS complex during pacing, suggesting aberrant intraventricular electrical conduction (Figure 1D). Fractional shortening was marginally decreased from 44.2 ± 2.0% at baseline to 37.2 ± 2.8% during pacing (p < 0.05; Table 1).

**Table 1:** Echocardiographic Measurements before Initiation of Pacing and During Pacing in Wildtype C57BL/6J Mice

	Pre-Pacing (n = 6)	During Pacing (n = 6)
IDD, mm	3.8 ± 0.14	3.6 ± 0.08
IDS, mm	2.1 ± 0.10	2.2 ± 0.12
Fractional Shortening, %	44.2 ± 2.0	37.2 ± 2.8*
AWTs, mm	1.1 ± 0.14	0.85 ± 0.08
AWTd, mm	0.53 ± 0.09	0.47 ± 0.05
PWTs, mm	1.4 ± 0.14	1.2 ± 0.10
PWTd, mm	1.0 ± 0.11	0.85 ± 0.13

Data is presented as group means ± SEM. Comparisons between groups were performed with unpaired T-tests. *, p < 0.05. IDD, IDS, intraventricular dimensions at end-diastole, end-systole; AWTs, AWTd, anterior wall thicknesses at end-systole, end-diastole; PWTs, PWTd, posterior wall thicknesses at end-systole, end-diastole.

Although global ventricular function was slightly diminished during pacing, fractional shortening was not significantly altered after cessation of pacing in the paced mice compared to matched sham-paced controls or compared to their baseline values. Similarly, left ventricular dimensions and wall thicknesses were not significantly changed in paced mice compared to matched sham-paced controls or baseline values (Table 2). Thus, short-term pacing was associated with dyssynchrony during pacing and a slight decrement in ventricular function that normalized immediately after cessation of pacing.

**Table 2:** Echocardiographic Measurements in C57BL/6J Wildtype Mice after Cessation of Short-Term Pacing

	Sham-Paced, 6 hr (n = 6)	Paced, 6 hr (n = 6)
IDD, mm	3.1 ± 0.13 (0.3 ± 0.1)	3.2 ± 0.18 (0.1 ± 0.2)
IDS, mm	1.8 ± 0.12 (0.2 ± 0.1)	1.9 ± 0.12 (0.1 ± 0.2)
Fractional Shortening, %	43.8 ± 1.7 (-2.3 ± 1.7)	40.6 ± 2.4 (-1.1 ± 1.2)
AWTs, mm	1.2 ± 0.09 (-0.13 ± 0.13)	1.2 ± 0.12 (0.03 ± 0.08)
AWTd, mm	0.8 ± 0.07 (-0.07 ± 0.1)	0.8 ± 0.1 (0.07 ± 0.1)
PWTs, mm	1.6 ± 0.03 (-0.43 ± 0.08)	1.4 ± 0.16 (-0.43 ± 0.29)
PWTd, mm	1.0 ± 0.05 (-0.75 ± 0.12)	1.0 ± 0.13 (-0.42 ± 0.18)

Post-pacing data (and changes from pre-pacing baseline) are presented as group means ± SEM. Comparisons between groups were performed with unpaired T-tests. IDD, IDS, intraventricular dimensions at end-diastole, end-systole; AWTs, AWTd, anterior wall thicknesses at end-systole, end-diastole; PWTs, PWTd, posterior wall thicknesses at end-systole, end-diastole.

### Short-Term Pacing Results in Focal Reduction of Cx43 Immunosignal Area at the Endocardium of the Left Ventricular Free Wall

**Figure 2:**
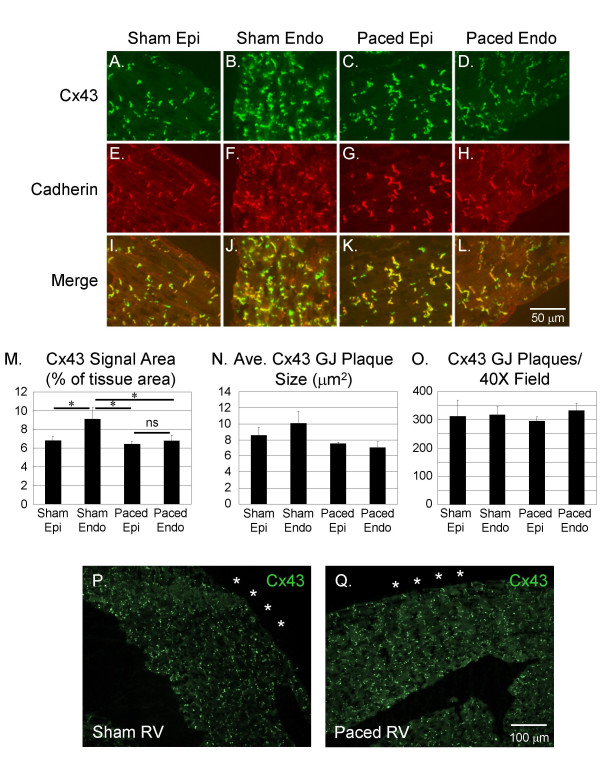
**Immunofluorescence Images of Cx43 and Cadherin Staining in the Epicardial and Endocardial Regions of Sham-Paced and Paced Hearts**. Cx43 immunosignal area is higher in the endocardium than the epicardium in sham-paced hearts (Panels A and B). In the paced hearts, Cx43 immunosignal area is decreased specifically at the endocardium, thereby eliminating the gradient of endocardial to epicardial Cx43 immunosignal area seen in the sham-paced hearts (Panels C and D). Cadherin staining pattern and area is unchanged in the paced hearts (Panels E-H). Co-localization of Cx43 and cadherin immunosignal, as demonstrated by the merged images, is statistically unchanged in the paced hearts compared to sham-paced controls (Panels I-L). Quantification of Cx43 immunosignal area shows an increasing epicardial-to-endocardial gradient in the sham but not in the paced mice, due to decreased signal area specifically in the epicardial region of the paced mice (Panel M). Average Cx43 gap junction (GJ) plaque size appears to increase from epicardium to endocardium in sham but not paced mice, although these differences were not statistically significant (Panel N). There were no significant differences in the number of Cx43 GJ plaques per high power field in epicardial vs. endocardial segments of sham and paced mice (Panel O). Cx43 immuno-staining pattern appeared similar at the RV apex of sham (Panel P) and paced hearts (Panel Q). RV epicardial surface is denoted by asterisks. Epi, epicardial region; Endo, endocardial region.

To characterize the effect of short-term pacing-induced dyssynchrony on gap junction distribution in the mouse heart, we paced mice for 6 hours (3–4 half-lives of Cx43) [24-26] and assessed Cx43 localization using immunofluorescence staining. In sham-paced mice, Cx43 immunosignal was arrayed in heterogeneously distributed membrane-associated plaques throughout the myocardium (Figure 2A and 2B). In the sham-paced hearts, Cx43 signal area as a percentage of total tissue area was greater in the endocardial region of the left ventricular (LV) free wall (9.12 ± 1.20%) than in the epicardial region (6.82 ± 0.40%; p < 0.05; Figure 2M). After 6 hours of pacing, however, Cx43 immunosignal area was reduced specifically at the endocardial region of the LV free wall (6.76 ± 0.60%) in comparison to the endocardium of sham-paced controls (p < 0.05), without a detectable change in epicardial Cx43 signal (6.41 ± 0.30%; Figure 2C and 2D and Figure 2M; data are from n = 6 sham and 6 paced hearts).

Post-hoc analysis showed that the Cx43 signal area was decreased significantly only at the basal LV endocardial segments in paced hearts compared to sham-paced hearts (6.38 ± 0.50% in paced vs. 9.36 ± 1.28% in sham, p < 0.01). There were trends at the apical (6.76 ± 0.89% in paced vs. 7.97 ± 0.96% in sham) and mid-ventricular (7.15 ± 0.69% in paced vs. 9.10 ± 1.59% in sham) LV endocardial segments toward decreased Cx43 signal area in paced hearts, although these were not statistically significant.

There was no difference in Cx43 signal area between sham (3.40 ± 0.25%) and paced (3.91 ± 0.45%) RV sections. Specifically at the RV apex where the stimulating electrode was placed, there was no statistically significant difference in Cx43 signal area between sham (3.51 ± 0.46%) and paced hearts (4.16 ± 0.53%; Figure 2P and 2Q).

Trends in endocardial gap junction plaque sizes mirrored changes in Cx43 immunosignal area, although differences in plaque size among groups were not statistically significant (Figure 2N). There was no significant difference in the number of gap junction plaques per 40× field in sham and paced endocardial and epicardial regions (Figure 2O). Thus, the epicardial-to-endocardial gradient of increasing Cx43 signal area observed in the LV free wall of sham-paced hearts, which has been described previously by other investigators[17], was eliminated after 6 hours of pacing due to a focal decrease in Cx43 immunosignal area at the endocardium of the LV free wall.

### Relationship of Cx43 to Cardiac Adherens Junctions is Unaffected by Short-Term Pacing

Adherens junctions, like gap junctions, are concentrated at the intercalated discs of adult cardiac myocytes. Since the expression pattern of Cx43 immunosignal was altered by short-term pacing, we investigated whether immunosignal area of cadherin, a critical component of the adherens junction with a half life of 5–6 hours [27,28], was also changed. Unlike the epicardial-to-endocardial gradient of increasing Cx43 immunosignal area, there was no difference in cadherin immunosignal area between epicardial and endocardial regions in sham-paced control hearts (Figure 2E and 2F). Furthermore, cadherin signal area in both epicardial and endocardial regions remained unchanged after 6 hours of pacing (p = NS; n = 6 sham and 6 paced hearts; Figure 2G and 2H). This suggests that the reduction in endocardial Cx43 signal area with pacing occurs in the absence of changes in the distribution of the adherens junctions.

To determine whether short-term pacing was associated with redistribution of Cx43 signal away from the intercalated disc, we performed colocalization analysis of Cx43 and cadherin immunosignal (Figure 2I–L). The percentage of Cx43 immunosignal colocalizing with cadherin in the paced hearts was unchanged in comparison to sham-paced controls. This suggests that short-term pacing in the mouse heart does not affect the process whereby Cx43 protein is directed to sites of adherens junction aggregation in the sarcolemma [29].

To further assess the effect of short-term pacing on targeting of Cx43 protein along the sarcolemma, we used a subjective scoring system in which the degree of Cx43 immunosignal at the lateral myocyte borders in sections from paced and sham-paced hearts was compared. Paced and sham heart sections were blindly assigned scores ranging from 0 (no lateralization) to 3 (extensive lateralization). We found that sham sections demonstrated extensive subjective lateralization of Cx43 signal (2.75 ± 0.25) and that the extent of lateralization did not appear significantly different in the paced hearts (2.40 ± 0.40, p = NS; n = 4 sham and 5 paced).

### Expression of Cx43 mRNA is Down-Regulated in the LV Free Wall after 6 hours of Pacing

**Figure 3:**
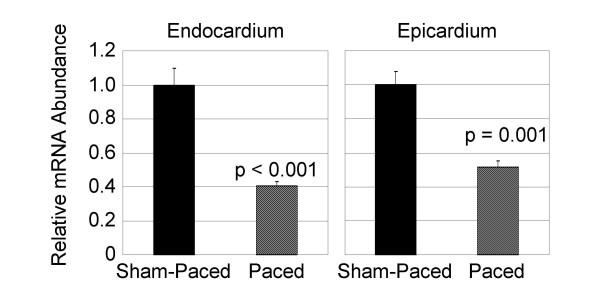
**Quantitative Real-Time PCR (qRT-PCR) Demonstrates Decreased Cx43 mRNA Levels in Paced Hearts**. Both the endocardium and the epicardium of the LV free wall demonstrated significantly reduced Cx43 mRNA levels after the short-term pacing protocol.

Since Cx43 immunosignal area was reduced at the LV endocardium after 6 hours of pacing, we investigated the effects of short-term pacing on Cx43 mRNA levels in the LV free wall endocardial and epicardial regions of paced and sham-paced control hearts. As shown in Figure 3, qRT-PCR demonstrated a 2.5 ± 0.1-fold down-regulation in expression of Cx43 mRNA at the endocardial region of the paced hearts compared to the sham-paced group (p < 0.001; n = 3 sham and 3 paced). Furthermore, a 2.0 ± 0.1-fold reduction in Cx43 mRNA expression was observed at the epicardial regions of the paced hearts compared to sham-paced controls (p = 0.001; n = 3 sham and 3 paced). Thus, six hours of pacing at rates just above those of sinus rhythm resulted in significant reductions in Cx43 mRNA levels at the LV free wall.

### Cx43 Abundance is Preserved in the LV Endocardial Region after Short-Term Pacing

**Figure 4:**
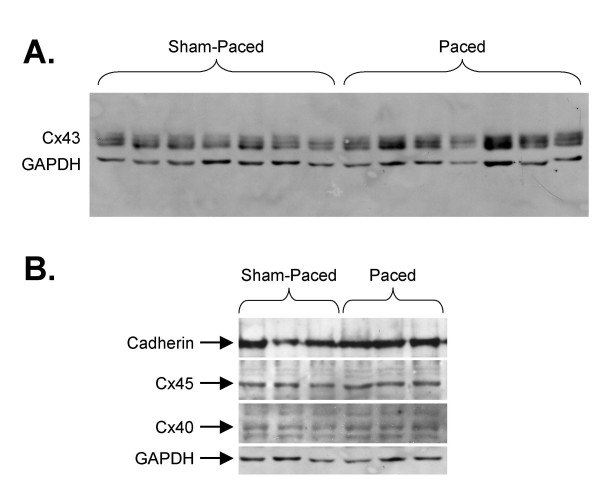
**Cx43, Cx45, Cx40 and Cadherin Protein Levels are Unchanged in Paced Hearts**. **A) **Cx43 protein abundance in the paced hearts was unchanged compared to sham-paced controls. **B) **Immunoblotting for cadherin, Cx45 and Cx40 reveals no significant differences in the mean band densities of these proteins normalized to GAPDH in the paced endocardial lysates compared with samples from sham-paced hearts.

Since short-term pacing in the murine heart resulted in down-regulation of Cx43 mRNA levels in the LV and decreased immunosignal of Cx43 at the endocardium, we investigated whether endocardial Cx43 protein abundance was affected by pacing. Surprisingly, there was no significant change in Cx43 protein expression in the paced LV endocardial region compared to sham-paced controls (n = 11 sham and 11 paced; Figure 4A). Densitometry values of the slower migrating (upper) Cx43 bands expressed as a ratio to the lower-most band was unchanged in paced compared to sham-paced samples, suggesting that the overall phosphorylation status of Cx43 was unaffected by short-term pacing. Expression levels of cadherin, Cx40 and Cx45 by immunoblotting were also statistically unchanged after pacing in the LV free wall endocardial myocardium (n = 7 sham and 7 paced; Figure 4B). Thus, despite significant reductions in Cx43 immunosignal area and mRNA abundance in the paced hearts, overall Cx43 protein expression levels are preserved.

### Altered Distribution of Cx43 in Paced Hearts

**Figure 5:**
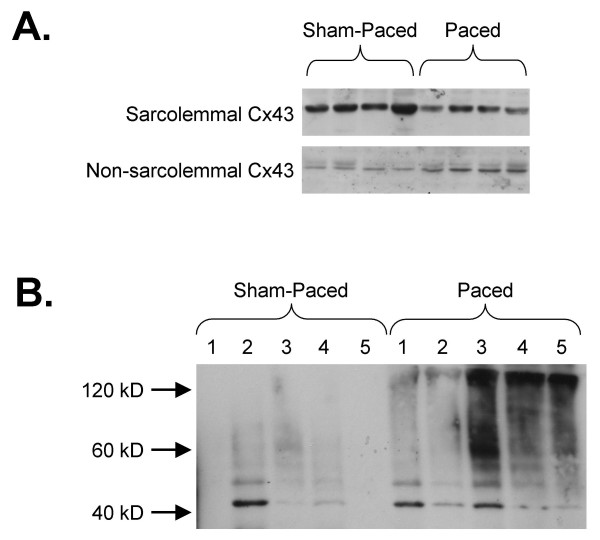
**Alterations in Cx43 Distribution and Ubiquitination with Pacing**. **A) **Fractionation of heart samples suggests that Cx43 is reduced in the sarcolemma-enriched fraction of the paced hearts and increased in the non-sarcolemmal supernatant. **B) **Immunoprecipitation for Cx43 and blotting for ubiquitin suggests that there is an increased accumulation of ubiquitinated Cx43 in the paced hearts compared to sham-paced controls. In this image, mono-ubiquitinated Cx43 is seen just above the 40 kD size marker and poly-ubiquitinated forms of Cx43 appear as a slower migrating smear.

Since total endocardial Cx43 protein levels in the paced hearts were unchanged despite reduced mRNA levels and immunosignal area, we considered that pacing might have resulted in redistribution of Cx43 from the sarcolemma into non-sarcolemmal pools. Fractionation of heart samples demonstrated significantly reduced Cx43 abundance in the sarcolemma-enriched fraction isolated from paced hearts compared with sham-paced controls (40.4 ± 8.7% decrease in the paced hearts; p < 0.05; n = 11 sham and 12 paced hearts; Figure 5A). In contrast, Cx43 levels in the supernatant (non-sarcolemmal fraction) increased by 104.0 ± 35.5% (p < 0.05; Figure 5A). These findings suggest that the intracellular distribution of cardiac Cx43 may be altered by short-term pacing with a reduced proportion that is localized to the sarcolemma.

With reduced Cx43 mRNA levels despite preserved total protein abundance and an apparent redistribution of Cx43 immunosignal from the sarcolemma, we predicted that the process of degradation of gap junction moieties might be disrupted, resulting in an accumulation of ubiquitinated Cx43 in the paced hearts. We tested this prediction by immunoprecipitating Cx43 in lysates from five sham-paced and five paced hearts, followed by gel electrophoresis and blotting for ubiquitin. We observed a substantially increased expression of ubiquitinated forms in the paced hearts (Figure 5B). These findings suggest that while Cx43 immunosignal area and mRNA levels are decreased, overall Cx43 protein levels in the paced hearts are preserved, possibly due to a combination of decreased production, altered intracellular distribution and disrupted degradation of Cx43.

### Electrocardiographic and Electrophysiologic Parameters are Unaltered after Short-Term Pacing

We next investigated whether pacing-induced changes in Cx43 expression were associated with persistent alterations in electrocardiographic and electrophysiologic indices. After cessation of pacing, we did not detect significant changes in electrocardiographic parameters in the paced mice when compared with baseline values or sham-paced controls (Table 3). There was also no significant difference between paced and sham-paced mice in refractoriness as determined by PES after the pacing period (Table 3). Furthermore, PES with single and double extrastimuli did not result in sustained ventricular arrhythmias in any of the mice tested. Thus, there was no effect of short-term pacing at rates just above sinus in wild type mice on electrocardiographic or electrophysiologic parameters. These data suggest that the observed pacing-induced changes in Cx43 distribution are not sufficient, in the absence of other insults to the myocardium, to cause a demonstrable arrhythmogenic effect.

**Table 3:** Electrocardiographic Indices and Electrophysiologic Data in C57BL/6J Wildtype Sham and Paced Mice

	Sham-Paced, 6 hr	Paced, 6 hr
QRS Duration, ms	11.1 ± 0.7 (-1.3 ± 0.7)	14.1 ± 1.0 (0.9 ± 1.1)
RR Interval, ms	144.6 ± 8.0 (12.6 ± 8.3)	144.6 ± 4.7 (0.9 ± 7.0)
QTc, ms	97.7 ± 4.2 (-6.7 ± 5.7)	106.2 ± 2.3 (0.6 ± 4.1)
VERP_100_, ms	34.3 ± 4.5	34.2 ± 3.0
VERP_80_, ms	36.3 ± 4.3	35.0 ± 3.6

Post-pacing data (and changes from pre-pacing baseline) are presented as group means ± SEM. Comparisons between groups were performed with unpaired T-tests. For electrocardiographic indices, n = 8 sham and 14 paced; for electrophysiologic data (VERP), n = 5 in each group. VERP, ventricular effective refractory period.

## Discussion

In this study, we investigated the effects of short-term, single-lead ventricular pacing on Cx43 expression and distribution. We found that pacing at the epicardial surface of the RV using the subdiaphragmatic approach was associated with dyssynchronous systolic contraction of the left ventricle. Pacing for only six hours at rates within 10% – 15% of sinus rhythm resulted in significantly reduced membrane-based Cx43 immunosignal in the endocardial regions of the LV free wall. While levels of Cx43 mRNA were significantly decreased, Cx43 protein abundance was unchanged by pacing in lysates isolated from the endocardial third of the LV free wall. Fractionation of ventricular lysates was consistent with a partial redistribution of Cx43 protein from the sarcolemma into non-sarcolemmal pools in the paced hearts compared to sham-paced controls. Immunoprecipitation of Cx43 and blotting for ubiquitin demonstrated a substantially increased accumulation of ubiquitinated Cx43 in the paced hearts. Thus, our studies suggest that short-term pacing results in significant reductions in Cx43 transcription and/or mRNA stability, altered intracellular distribution of gap junction protein, and a disruption in the process of degradation of Cx43.

It is intriguing that in the absence of an underlying disease process, such as tachycardia-induced cardiomyopathy, hypertrophy or ischemia, short-term pacing of the heart at rates just above that of normal sinus rhythm should produce significantly decreased Cx43 mRNA levels and intracellular redistribution of Cx43 protein. Nevertheless, our observations are in keeping with findings from pacing studies in other animal models. Patel, et al, performed a study in which dogs were paced for 21 days at rates 10% – 15% above the normal sinus rate [11], in contrast to canine models of pacing-induced heart failure in which the ventricles are paced at more than twice the normal sinus rate [30-32]. Patel and colleagues observed an apparent redistribution of Cx43 labeling from the intercalated discs to the lateral cell membranes of myocytes close to the site of pacing. This redistribution of Cx43 immunosignal is reminiscent of the gap junction remodeling seen in the peri-infarct zone of experimental myocardial infarction [6]. However, acute pacing such as that performed in the present study may be insufficient to cause gap junction remodeling of the extent seen in ischemic conditions.

In another model of the effects of chronic dyssynchrony, dogs studied four weeks after radiofrequency ablation of the left bundle branch were found to have Cx43 lateralization that was associated with reductions in conduction velocity, action potential duration and refractory period [10]. Lateralization of Cx43 signal and the accompanying electrophysiologic alterations in the canine left bundle branch block model were limited to epicardial regions in the late-activated lateral wall of the left ventricle. Altered stress-strain relationships during ventricular pacing have been observed previously using magnetic resonance imaging [33,34]. Hence, it is conceivable that altered stress-strain relationships, owing to intraventricular and interventricular dyssynchrony, may be important factors underlying altered intracellular gap junction distribution in the left bundle branch block model, as well as in our model of epicardial right ventricular pacing.

While altered gap junction expression was observed after several weeks of pacing or experimentally induced left bundle branch block in the canine model, other data suggest that changes in gap junction expression may be induced over a much shorter time frame. Ischemia induced by cessation of perfusion for up to 40 min in the isolated rat heart resulted in dephosphorylation and redistribution of Cx43 from the intercalated disc, without a net loss of Cx43 protein abundance from the myocardium. Lateralized Cx43 immunosignal in the isolated ischemic rat heart appears to be mainly phosphorylated in those hearts that recovered contractile function, but nonphosphorylated in those hearts that did not recover function during reperfusion [12]. Based on these data, we predicted that short-term pacing in the mouse may also result in redistribution of Cx43 signal within the ventricular myocyte.

The effect of short-term pacing on connexin expression has been previously described in the setting of strong electric currents, which were up to 100 times greater than those used in this study and represented a model of cardiac tissue damage [21]. In that study, Sambelashvili et al. provided fundamental insight into cardiac stimulation by demonstrating the effect of acute tissue damage on virtual electrode polarization patterns. In addition, they observed changes in local connexin expression near the stimulating electrode under control conditions (10 mA) and "damage" conditions (40 mA). The extent of connexin loss was greater under damage conditions suggesting a voltage dose-response. Our study differs in that we used the minimum current density (= 0.4 mA) to ensure sustained capture. Our intention was to model altered activation rather than acute tissue damage. Interestingly, our exhaustive studies of tissue near the site of stimulation did not reveal local changes in connexin expression. However, we did observe changes that were distant and more diffuse, yet more subtle.

Our results demonstrating a gradient of increasing epicardium to endocardium Cx43 immunostaining area in the sham-paced hearts are consistent with data presented by other groups. Yamada et al. observed a similar transmural gradient of Cx43 immunosignal distribution in mice of the same strain as that used for our study [17]. A transmural gradient of Cx43 expression in the canine heart has been associated with gradients in conduction velocity and action potential duration [32]. These data suggest that regional heterogeneities in the expression of Cx43 may underlie important electrophysiologic properties of the heart. In this study, we have observed that short-term pacing at rates just over that of sinus rhythm eliminates the transmural gradient of Cx43 immunosignal area. While the normally functioning heart may be able to absorb subtle alterations in the distribution of Cx43, such as those induced by short-term pacing, in the setting of additional insults similar changes in Cx43 distribution may result in more deleterious electrophysiologic consequences.

Previously, we and others have observed significant changes in ECG parameters and a substantially elevated risk of lethal ventricular tachyarrhythmias in conditional models of decreased cardiac Cx43 expression [9,35-37]. In the present study, we have observed no persistent changes in the ECG and no increased inducibility of arrhythmias after cessation of pacing despite significant intracellular redistribution of Cx43 protein. Clearly, the magnitude of altered Cx43 localization due to short-term pacing in and of itself is insufficient to result in significant arrhythmogenicity. In the setting of reduced baseline Cx43 expression, however, such as we and others have described in the diseased heart [3-6], or in combination with superimposed electrophysiologic changes due to ischemia, pacing-induced gap junction remodeling may have significant arrhythmic implications. Furthermore, our present findings do not rule out the possibility that more prolonged pacing might promote additional changes in Cx43 expression and distribution that would have potentially pro-arrhythmic effects.

The Cx43 gap junction is known to be degraded along the ubiquitin proteasome pathway, with involvement of the endosome/lysosome either sequentially or in parallel [38,39]. Ubiquitination and subsequent degradation of Cx43 is a highly regulated process, in which epidermal growth factor (EGF) may play a prominent role [40,41]. However, while EGF-induced ubiquitination of Cx43 is associated with hyperphosphorylation of the gap junction protein, we did not detect a change in the phosphorylation status of Cx43 in the paced endocardial myocardium. This suggests that the ubiquitinated forms of Cx43 may accumulate in the paced hearts in a process independent of EGF.

We intend to use the pacing model to broaden our understanding of the role of Cx43 and its regulation in cardiac disease. However, an important potential limitation of this model is that the mechanisms underlying pacing-induced changes in gap junction expression are not necessarily the same as those responsible for gap junction remodeling observed in ischemia, the peri-infarct zone or other pathological states. Nonetheless, defining the mechanisms responsible for pacing-induced alterations in the intracellular distribution of Cx43 will allow us to generate hypotheses that will be testable in models of cardiac disease.

## Conclusion

In summary, we report that short-term cardiac pacing at rates just fast enough to ensure capture, induces mechanical dyssynchrony of the left ventricle, significantly decreased Cx43 mRNA levels and partial redistribution of Cx43 protein in the murine heart. These data suggest that limited exposure to dyssynchronous activation results in remodeling of the cardiac gap junctions in the absence of sustained measurable effects on contractility or arrhythmic inducibility. However, in the setting of cardiac disease with decreased baseline Cx43 expression, dyssynchronous activation with its attendant effects on gap junction remodeling may further exacerbate arrhythmic complications and worsen cardiac performance.

## Authors' contributions

AK participated in the design of the study, carried out or assisted in all experiments and drafted the manuscript. RAK participated in the design of the study, carried out pacing, immunoblotting and immunofluorescence experiments and helped to draft the manuscript. EK carried out immunoblotting experiments and helped to draft the manuscript. JEF carried out the qRT-PCR experiments. PPG carried out the immunoprecipitation experiments. MP carried out the lysate fractionation experiments. FL carried out immunofluorescence experiments. MJR helped design, oversee and interpret the lysate fractionation and immunoprecipitation experiments. ALW helped conceive of the study, participated in its design and helped to draft the manuscript. EAF participated in the design of the study and coordinated the qRT-PCR experiments. NSP helped conceive of the study, participated in its design and helped to draft the manuscript. DEG conceived of the study, participated in its design, oversaw coordination and execution of the study and drafted the manuscript. All authors have read and approved the final manuscript.
